# Dermatitis Herpetiformis: Novel Perspectives

**DOI:** 10.3389/fimmu.2019.01290

**Published:** 2019-06-11

**Authors:** Emiliano Antiga, Roberto Maglie, Lavinia Quintarelli, Alice Verdelli, Diletta Bonciani, Veronica Bonciolini, Marzia Caproni

**Affiliations:** Section of Dermatology, Department of Health Sciences, University of Florence, Florence, Italy

**Keywords:** dermatitis herpetiformis, epidermal transglutaminase, coeliac disease, non-coeliac gluten sensitivity, direct immunofluorescence

## Abstract

Dermatitis herpetiformis (DH) is an inflammatory disease of the skin, considered the specific cutaneous manifestation of celiac disease (CD). Both DH and CD occur in gluten-sensitive individuals, share the same Human Leukocyte Antigen (HLA) haplotypes (DQ2 and DQ8), and improve following the administration of a gluten-free diet. Moreover, almost all DH patients show typical CD alterations at the small bowel biopsy, ranging from villous atrophy to augmented presence of intraepithelial lymphocytes, as well as the generation of circulating autoantibodies against tissue transglutaminase (tTG). Clinically, DH presents with polymorphic lesions, including papules, vesicles, and small blisters, symmetrically distributed in typical anatomical sites including the extensor aspects of the limbs, the elbows, the sacral regions, and the buttocks. Intense pruritus is almost the rule. However, many atypical presentations of DH have also been reported. Moreover, recent evidence suggested that DH is changing. Firstly, some studies reported a reduced incidence of DH, probably due to early recognition of CD, so that there is not enough time for DH to develop. Moreover, data from Japanese literature highlighted the absence of intestinal involvement as well as of the typical serological markers of CD (i.e., anti-tTG antibodies) in Japanese patients with DH. Similar cases may also occur in Caucasian patients, complicating DH diagnosis. The latter relies on the combination of clinical, histopathologic, and immunopathologic findings. Detecting granular IgA deposits at the dermal-epidermal junction by direct immunofluorescence (DIF) from perilesional skin represents the most specific diagnostic tool. Further, assessing serum titers of autoantibodies against epidermal transglutaminase (eTG), the supposed autoantigen of DH, may also serve as a clue for the diagnosis. However, a study from our group has recently demonstrated that granular IgA deposits may also occur in celiac patients with non-DH inflammatory skin diseases, raising questions about the effective role of eTG IgA autoantibodies in DH and suggesting the need of revising diagnostic criteria, conceivably emphasizing clinical aspects of the disease along with DIF. DH usually responds to the gluten-free diet. Topical clobetasol ointment or dapsone may be also applied to favor rapid disease control. Our review will focus on novel pathogenic insights, controversies, and management aspects of DH.

## Introduction

About 135 years ago, the American dermatologist Luis Duhring reported for the first time an itching and multiform skin eruption with erythema, papules, bullae, pustules, and above all, grouped vesicles localized at typical areas (1). At that time, he had seen 15 cases of such a disease, and he decided to name it “dermatitis herpetiformis,” due to its clinical similarity to herpes virus infection (1). After the identification of dermatitis herpetiformis, several years passed before the second important step in its early story until 1950, when dapsone was accidentally found to be effective for the treatment of the disease (2). Then, 50 years ago, Van der Meer described the typical IgA deposits at the papillary tips, which represent the immunological hallmark of DH as well as the clue for the diagnosis of the disease (3).

However, it was only in the 60s and, above all, in the 70s that DH was clearly related to gluten intolerance, allowing to consider the disease as the specific cutaneous manifestation of celiac disease (CD). In fact, patients with DH were shown to have in most cases intestinal changes similar to those found in CD (4) and to share the same genetic background of CD (5), and gluten-free diet (GFD) was demonstrated to improve skin symptoms (6), although more slowly than the gastrointestinal ones.

Since then, several issues about the pathogenesis and the management of DH have been pointed out (7). Among them, the identification of reliable serologic markers of the disease (8) and the discovery of epidermal transglutaminase (eTG) as the autoantigen of DH were some of the most remarkable steps (9).

DH can be defined as a disease presenting with: (i) symmetrical polymorphic lesions involving typical areas such as the extensor aspects of the limbs and sacral region, (ii) a predominant neutrophilic infiltrate at the dermal papillae at histopathology, (iii) granular IgA deposits along the dermal-epidermal junction, (iv) an invariable association with CD, and (v) a response to a lifelong GFD (7).

However, despite the growing knowledge about the disease, several issues have to be clarified yet, and some of the cornerstones of DH need further discussion. For example, the specificity of IgA deposits in the perilesional skin of patients with DH could be questioned and the diagnostic algorithm should be revised (10), taking also into account the identification of non-coeliac gluten sensitivity (NCGS) as a new entity among the spectrum of gluten-related disorders (11); moreover, the pathogenic role of IgA anti-eTG antibodies needs further demonstration, since they may also occur in patients without DH (12). Finally, even the therapeutic role of GFD is discussed, since some authors suggest that it can be interrupted in the (rare) case of DH remission (13).

The current review will try to address or at least to focus on these and other issues that are still under debate in the context of DH. The picture that hopefully will come out would be that of a lively disease that represents both a pathogenic model of autoimmunity and a paradigmatic example of how the skin can be the mirror of an internal condition such as CD.

## The Epidemiology of Dermatitis Herpetiformis: a (Rare) Disease of the Western Countries

DH is a rare disease that occurs prevalently in Caucasian individuals. In Europe and USA, the prevalence of DH ranges from 11.2 to 75.3 per 100.000, with the highest reported in Finland; whereas, the incidence ranges from 0.4 to 2.6 per 100.000 people per year (14–16). DH is extremely rare among African and Asian populations. Reasons explaining the low prevalence of DH among such populations include the absence of DH predisposing human leukocyte haplotypes (HLA) DQ2 and DQ8, which are always found in Caucasian DH patients, and the low wheat consumption in these geographic areas (17). However, in the Asians, including Chinese and Japanese populations, the association between DH and CD seems to be weaker than in Western countries, despite DH presents with similar clinical and immunopathological features. The extreme rarity of CD in these populations may have led, indeed, to overlook the diagnosis of DH and possibly underestimate the prevalence of the disease (17, 18).

During the last decades, the overall incidence of DH is significantly decreased, although the incidence of CD is increasing (19–22). One plausible explanation for the opposite trends in the epidemiology of CD and DH could be the increased awareness of CD among physicians and patients and the wide prescription of CD screening tests even in patients without typical gastrointestinal manifestations, leading to identify early patients with latent or potential CD.

DH can occur at any age, but typically occurs during adulthood, and mostly between the third and the fourth decade of life (14). Interestingly, in one Finnish study including 477 patients diagnosed with DH over a 40 year period, the patients' age at DH diagnosis was shown to significantly increase over time. This is presumably related to a parallel decrease of the annual consumption of wheat during the same period, leading to a lower lifetime gluten load (16).

DH has been also reported to occur in pediatric patients, but the exact incidence of DH during childhood is unknown. In one study by our group in 2013 including 159 DH patients, about 36% were diagnosed below the age of 20 (23). Other authors have suggested a possible underestimation of pediatric DH due to clinical overlapping features with atopic dermatitis, which still accounts for the most prevalent dermatologic disease among children (24). Unlike CD, DH seems to occur most commonly in man, although the male/female ratio has reportedly ranging from 2:1 to 1:1 according to different studies (16, 19, 24). Interestingly, a Finnish study found that in females there is a longer diagnostic delay compared to male (25).

### Associated Diseases

Like CD, DH has been found to be associated with several autoimmune disorders, including type I diabetes mellitus, autoimmune thyroid diseases, and connective tissue diseases, such as Sjögren syndrome (24). Of interest is also the reported association between DH and bullous pemphigoid (BP), a subepidermal autoimmune blistering disease characterized by autoimmunity against the hemidesmosomal antigens BP180 and BP230 (26, 27). Accordingly, in a retrospective case-control study, diagnosis of DH was found to increase of 22 folds the risk of BP, compared to only a 2-fold higher risk of developing BP amongst coeliac patients. The mean time between the diagnosis of DH and BP development was about 3 years (27).

Since virtually all patients with DH have CD, DH patients also carry an increased risk of non-Hodgkin lymphomas and gastrointestinal malignancies (28). However, unlike CD, mortality of DH patients is not increased (29–31). Accordingly, in a large population-based study including 476 patients with DH, a reduced mortality for all the causes of death compared to the general population and a significantly reduced mortality related to cerebrovascular diseases were shown. Mortality due to non-Hodgkin lymphomas was increased only during the first 5 years following the diagnosis but not thereafter. DH patients were also found to have less hypercholesterolemia, and there were fewer smoker compared to the control population (32). Ali and Lear speculated that smoking might have a protective effect against DH, since it suppresses natural killer (NK) lymphocytes and reduces intestinal IgA secretion (33–36). Finally, an association between DH and higher social class have been reported, and may contribute to the observed reduced mortality reflecting the “many facets of better living” of a high socioeconomic status (33).

### Clinical Manifestations

The polymorphism and the symmetrical distribution of the lesions represent the major clinical hallmarks of DH (37, 38).

**Figure 1:**
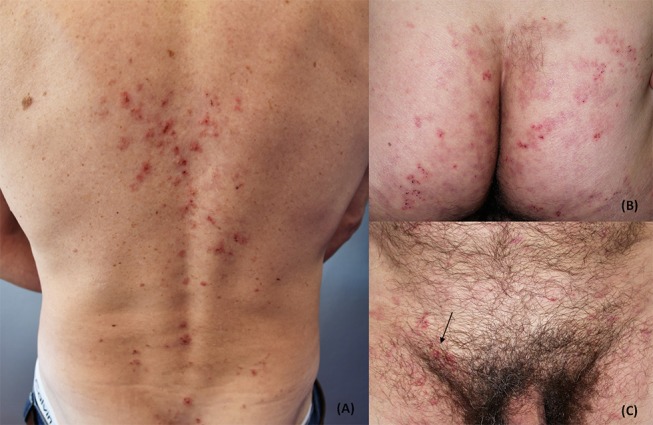
Clinical presentation of dermatitis herpetiformis (DH): erythematous grouped papules and vesicles associated with excoriations and crusts at the back **(A)**, sacral region and buttocks **(B)**. Rarely, DH may also affect the groin and pubis (arrow) **(C)**. The patient gave written informed consent for the publication of these pictures.

**Figure 2:**
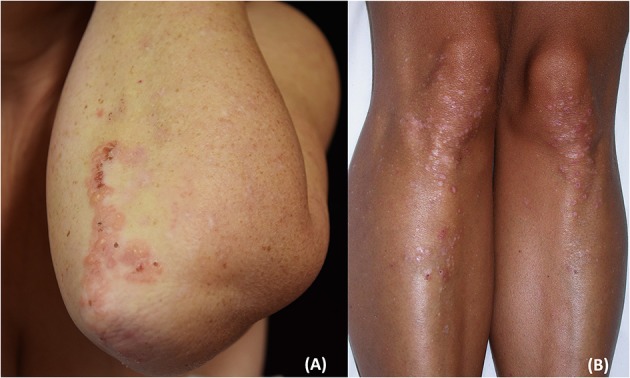
Clinical presentation of dermatitis herpetiformis: grouped papules and vesicles associated with excoriations and crusts at the elbows **(A)** and lower limbs **(B)**. Post-inflammatory pigmentary changes such as hypo-pigmentation could be also appreciated **(B)**. The patient gave written informed consent for the publication of these pictures.

The disease usually presents with grouped erythematous papules and urticarial plaques with overwhelming vesicles. The latter may then coalesce into small tense blisters with sero-hemorrhagic content, which are characterized by a centrifugal growth pattern. Erosions, excoriations, and crusts are likely to occur because of blisters rupture and because of the scratching secondary to the associated pruritus (15, 38, 39). Lesions eventually heal leaving post-inflammatory hypo- and hyper-pigmentation (Figures 1, 2).

Typically, DH lesions symmetrically localized at the extensor surfaces of upper and lower limbs, mostly at the elbows and knees, buttocks, and the sacral region; the abdomen, upper back, shoulders, nuchal area, and the scalp may also be involved, whereas the face and groin are rarely affected.

Likewise, mucosal involvement has sporadically been reported. Oral manifestations of DH mainly consist of erosions affecting both the oral mucosa and the tongue; associated symptoms include pain and burning sensation (15, 40). However, it is not clear whether oral involvement has to be considered as a specific manifestation of DH or, rather, a sign of the underlying CD; accordingly, oral aphthosis, erosions, and/or ulcerations are also frequently found in CD (40–44).

Pruritus is the leading symptom of DH and its absence is a strong argument against the diagnosis (45). In one study by our group including a cohort of 159 patients, almost all complained of severe pruritus, which had a significantly negative impact on the patient's quality of life. Moreover, in many cases, pruritus along with a stinging and burning sensation of the skin was shown to be the presenting sign of DH, preceding of 12–24 h the appearance of the cutaneous lesions (46). Notably, pruritus has reportedly occurred even months before the onset of skin lesions (47, 48).

### Atypical Cases of Dermatitis Herpetiformis: Are They Always Dermatitis Herpetiformis?

Several atypical cases of DH have been reported in the literature. Asymptomatic palmoplantar petechiae, occurring either alone or in association with characteristic DH clinical findings, have been reported in some cases of pediatric DH. Petechiae were found to occur prevalently at the dominant hand or foot, suggesting repeated microtraumatism as a possible trigger (44, 49–55).

Moreover, Naylor et al. reported a case of DH presenting with a diffuse petechial rush and microscopic changes consistent with both DH and vasculitis (56). Kern et al. reported a patient with DH presenting as pseudovasculitis, characterized by a diffuse petechial rush and a large ulcer at his extensor forearm, and histopathologic and direct immunofluorescence (DIF) findings consistent with DH without additional signs of small-vessel inflammation (57). A possible hypothesis of the association between DH and vascular damage might be the presence of perivascular IgA immune complexes leading to small vessel inflammation.

Finally, cases of DH presenting as palmoplantar keratosis (58), purpuric lesions with chronic urticaria (59), and prurigo pigmentosa-like lesions (60) have been also reported.

## Pathogenesis

**Figure 3:**
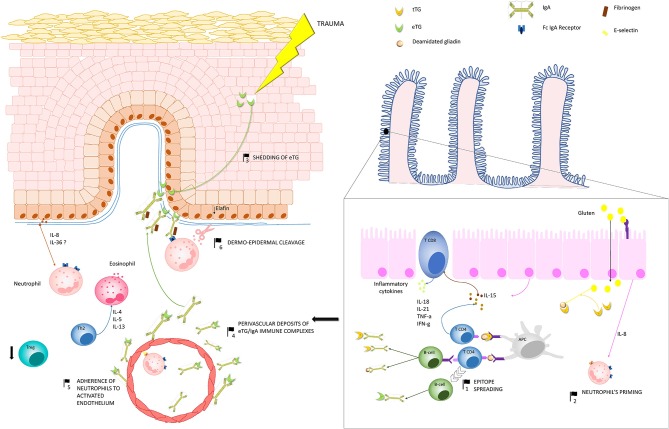
Pathogenesis of dermatitis herpetiformis (DH): Production of IgA autoantibodies against eTG occurs in the gut, probably as a result of an epitope spreading phenomenon (1), due to the high sequence homology between tissue TG, which is a major autoantigen in coeliac disease, and eTG. Activation of innate immunity in the gut leads to increased release of IL-8, which is thought to be responsible for the initial priming of neutrophils (2). One theory suggests that, in region of trauma, keratinocyte damage leads to shedding of eTG to the dermal-epidermal junction (3), where it binds to anti-eTG IgA. An alternative hypothesis suggests that eTG/IgA complexes exists as circulating immune complexes, which can deposit both at the dermal-epidermal junction and around dermal vessels (4). A complex interplay between inflammatory cytokines and the activation of fibrinogen stimulate neutrophil adherence to the activated endothelium (5) and migration to the dermal papillae. Herein neutrophils, which probably bind to IgA aggregates through the Fc IgA receptor, release proteases, which finally induce the cleavage of the dermal-epidermal junction (6). In parallel, hyper-activation of Th2 cells activate eosinophils, which also co-operate with neutrophils to the cleavage of the dermal-epidermal junction. Down-regulation of Treg cells do also occur, favoring the maintenance of a pro-inflammatory microenvironment in DH skin. Abbreviations in the figure: eTG, epidermal transglutaminase; tTG, tissue transglutaminase; IL, interleukin; Th, T helper cell; Treg, regulatory T cell; APC, antigen presenting cell.

DH represents a paradigmatic model of autoimmune disease, owing that it can be switched on or off by a known external trigger: the gluten. The pathogenesis of DH, which relies on a complex inflammatory network along the gut-skin axis, remains at present only partly understood. Over the past 30 years, major efforts have led to the identification of eTG as the main autoantigen of DH and to well-characterize the inflammatory microenviroment underlying skin lesions development. However, controversies still persist about the mechanisms by which (i) anti-eTG autoantibodies develop, (ii) they form the typical granular aggregates at the dermal papillary tips, and (iii) eventually induce the appearance of the skin lesions. Herein, we summarized the current knowledge of DH pathogenesis, highlighting the major controversial issues that may represent the starting point for future researches (Figure 3).

### HLA DQ2/DQ8 and Gluten Are Required for the Occurrence of Dermatitis Herpetiformis

About 5–10% of DH patients have a first-degree relative affected by either DH or CD (61–63), suggesting that genetic factors play a major pathogenic role in the disease. Previous studies have found an association between DH and both HLA class I and II molecules, including HLA A1, B8, DR3, and DPB1 (64). However, the closest association occurs with HLA-DQ2 (combination of the DQA1^*^0501 and DQB1^*^02 alleles) and DQ8 (combination of the DQA1^*^03 and DQB1^*^0302 alleles), which can be found in roughly 85 and 15% of the patients, respectively (65). Other studies have tried to investigate the role of more than 40 other non-HLA gene polymorphisms in the pathogenesis of both CD and DH, but none has yielded convincing results (66–69).

Both HLA-DQ2 and DQ8, whose genes map on the chromosome 6, are crucially involved in processing the gluten antigen gliadin (70), corresponding to the same predisposing haplotypes found in coeliac patients (71, 72). In CD, tissue TG (tTG) catalyze deamidation of gliadin, creating epitopes that increase gluten-peptide binding affinity to HLA-DQ2 and DQ8 expressed on the surface of antigen presenting cells, leading to an adaptive immune reaction against both tTG and gliadin. In parallel, activation of innate immunity leads to characteristic small bowel alterations, including reversible villous atrophy in the upper part of the jejunum, crypt hyperplasia on small bowel mucosal samples and prominent intraepithelial lymphocytosis (73–75). Virtually all DH patients show evidence of a potential or manifest, usually mild, CD, suggesting that DH represents a specific cutaneous manifestation of CD (76). The pathogenic relevance of HLA-DQ and gluten intake in DH has been further demonstrated experimentally by Marietta and coworkers. Firstly, they observed that, unlike other autoimmune blistering dermatoses, passive transfer of DH serum into athymic mice with human skin engrafts did not induce DH lesions. However, DH lesions developed in about 17% of HLA-DQ8 positive autoimmune prone NOD mice when they were exposed to gluten through periodic intraperitoneal injections. This suggests that DH develops only in the presence of predisposing HLA-DQ antigens together with gluten exposure (77, 78).

An exception to what aforementioned is DH occurring in Japanese populations. To date, only two studies have delineated the clinical and immunological features of Japanese DH, including a total of 116 patients, suggesting the extreme rarity of DH in Japan. Although these studies were biased by the fact that the results of some analyzed parameters, including the presence of villous atrophy, circulating antibodies, and response to a GFD, were available only for a few patients, some peculiar characteristics of Japanese DH have emerged, including (i) the absence of HLA DQ2/DQ8 haplotypes, (ii) the absence of an underlying CD, (iii) a higher clinical involvement of non-predilection sites such as the extremities and trunk, (iv) fibrillar rather than granular IgA deposits in the papillary dermis of a substantial proportion of patients, and (v) a lower incidence of CD-associated autoimmune diseases and non-Hodgkin lymphomas (Table 1) (18, 79). Even in some Chinese DH patients, the disease was shown to occur outside the setting of CD, which is also very rare in China (80). Altogether, these observations raise speculation on the existence of a subgroup of DH that may be not elicited by gluten, although showing some clinic-pathologic features like in gluten-related DH.

**Table 1:** Different characteristics between Caucasian and Japanese patients with dermatitis herpetiformis.

**Characteristics**	**Caucasian DH**	**Japanese DH**
HLA	HLA-DQ2 (DQB1^*^02:01)−85% HLA-DQ8 (DQB1^*^03:02)−15%	HLA-DQ2 (DQB1^*^02:01)−0% HLA-DQ8 (DQB1^*^03:02)−37%^*^
Sites of involvement	Elbow, buttock, knee, face, ear, neck, scalp, groin	Elbow, buttock, knee, face, ear, neck, scalp, groin Non-predilection sites, including the extremities and trunk. Whole body
Villous atrophy	Most of the patients	Not known^**^
Circulating anti-tTG IgA	50–95%	38%
Circulating anti-eTG IgA	50–95%	43%
DIF	Granular IgA deposits	Granular and fibrillar IgA deposits
Response to the GFD	Most of the patients	Lack of consistent data
Association with autoimmune diseases	Frequent	Rare

HLA, Human Leucocyte Antigen; DIF, Direct Immunofluorescent; GFD, gluten-free diet.

* *The frequency of HLA-DQ8 refers to the study by Ohata et al. (18), where the allele was found in six (37%) out of 16 Japanese patients with DH (19)*.

** *Found in three out of six patients in the study by Ohata et al. (79), including a total of 91 patients in 2012*.

### Triggers Other Than Gluten Possibly Involved in the Pathogenesis of Dermatitis Herpetiformis

There is evidence that external triggers other than gluten may induce or worsen DH. For example, potassium iodide, a common compound of expectorants, has reportedly been a trigger of DH both after oral ingestion and when applied topically (81). Similarly, Snider et al. described two cases of DH elicited by a cleaning solution (82).

Kovaleski et al. reported on two cases of DH following a gastric stapling procedure and a gastrectomy with partial pancreatectomy and colectomy, respectively (83). Similarly, a non-coeliac patient developing DH after a mini-gastric bypass surgery was reported (84). In these cases, the authors hypothesized that the surgery-induced enteral inflammation promoted a cross-reaction between cutaneous and intestinal anti-TG antibodies, that seems to be in accordance with the recent demonstration of the intestinal origin of anti-eTG IgA autoantibodies (85).

Hormonal factors, and specifically the hypothalamic-pituitary-gonadal axis, may play a role in DH. Accordingly, hormone replacement therapy for panhypopituitarism has been reported to cure DH (86). One study reported DH following progesterone contraception (87). Other two studies reported DH occurrence after a therapy with leuprolide acetate, an analog of the gonadotropin-releasing hormone (GnRH). In both cases, lesions resolved upon drug discontinuation. However, in one case, skin lesions recurred once the patient was started on biculatamide, another GnRH analog (88, 89).

Intriguingly, drugs may be also a potential trigger of DH. Marakli et al. described a patient who developed DH while on therapy with the Tumor Necrosis Factor-alfa inhibitor infliximab for an ankylosing spondylitis (90); whereas, Mochel et al. recently illustrated a patient who developed DH after several weeks of ipilimumab treatment for a metastatic melanoma. Interestingly, the patient had a positive result of anti tTG IgA antibodies prior to the diagnosis of the metastatic cancer but neither a confirmed diagnosis of coeliac disease nor active skin rush before taking ipilimumab (91). The latter is a monoclonal antibody targeting CTLA-4 and is thought to exert its anti-tumor activity by promoting T-cell activation and down-regulation of Treg function (92). It is arguable that the reported patient had a latent or silent gluten-sensitive enteropathy with an immunological reaction against tTG and eTG, and that inhibition of Tregs precipitated the manifestation of DH, in agreement with the critical role of Treg in the disease pathogenesis (93).

Finally, DH has reportedly been occurred close to a diagnosis of an adenocarcinoma of the lung and an autoimmune pancreatitis in two Japanese patients, who had neither serum IgA antibodies against eTG nor signs of an underlying CD (94).

Interestingly, a gastrointestinal infection appears to be an essential factor for the induction of CD. Several pathogenes have been suggested to trigger CD, including rotavirus, Epstein Bar Virus, Cytomegalovirus, HCV, HBV, *Bacteroides* species, *C. jejuni, Pneumococcus, M. tuberculosis*, and *H. pylori* (95). A recent study demonstrated a link between Reovirus, an avirulent pathogen that elicit protective immunity, and the loss of peripheral tolerance against dietary antigens, resulting in a Th1-type immunity to dietary antigens. Moreover, the study found an increased titer of antibodies against Reovirus in patients with active CD and elevated serum anti-tTG autoantibodies, suggesting a direct link between the pathogen and the induction of CD (96). Whether there might be an infectious trigger also for DH is far less clear (97).

To conclude, complex endocrine and immunologic factors seem to play a role in modulating the inflammatory response in DH, suggesting that its pathogenesis is much more complex than a simple interaction between HLA-DQ antigens and gluten.

### Epidermal Transglutaminase Is the Main Autoantigen of Dermatitis Herpetiformis

Epidermal transglutaminase (eTG) belongs to a nine-member Ca^2+^-dependent enzyme family that promotes the formation of covalent cross-links between proteins (98). eTG is physiologically expressed in the spinous layer of the epidermis, and contribute to epidermal terminal differentiation, formation of the cornified cell envelop, and protection of keratinocytes against UVB-induced apoptosis (99–102).

While tTG was shown to be a major autoantigen of CD, Sardy et al. identified eTG as the main autoantigen of DH (9). Specifically, they observed that CD and DH patients had autoantibodies targeting both tTG and eTG; however, IgA autoantibodies binding selectively and with high avidity to eTG were found only in DH patients. Moreover, eTG, but not tTG, was found to co-localize with IgA in the granular deposits at the papillary tips of DH skin (9). The mechanism by which both CD and DH patients develop an autoimmune response against eTG remains still obscure. One suggested hypothesis is related to epitope spreading (99). The phenomenon of epitope spreading involves the development over time of a humoral or cell-mediated immune response from an initial dominant epitope to a secondary one, belonging to the same (intramolecular) or a distinct (intermolecular) antigen (103). Evidence supporting the theory of epitope spreading in DH include: (i) the high sequence homology between tTG and eTG (9); (ii) the presence of an autoimmunity also against neuronal TG (or TG6), which is also highly similar to tTG and eTG, in both CD and DH (99); (iii) the lower prevalence of anti-eTG IgA autoantibodies in pediatric compared to adult CD patients, which (iv) parallels the decreased, albeit not abolished, incidence of DH during childhood (23, 104).

One recent study demonstrated that patients with active DH secreted considerably high amounts of anti-eTG IgA in the organ culture medium of small bowel mucosal biopsies, and had eTG-binding IgA-positive cells in the lamina propria, thereby suggesting that autoimmunity against eTG possibly develops in the gut (85). Interestingly, small bowel secretion of eTG-targeting IgA did not occur in CD patients, despite they showed elevated levels of such autoantibodies in the serum (85).

An unmet issue concerns the mechanism underlying the formation of eTG/IgA aggregates in the skin. One theory suggests that, in regions of trauma, where DH lesions classically occurs, epidermal damage leads to eTG shedding from the spinous layer to the upper dermis, where it binds to circulating anti-eTG IgA. A study by Zone et al. supports this hypothesis, showing that passive transfer of goat anti-eTG IgG or DH serum into mice with human skin grafts reproduced DH-like deposits only in the engrafted skin, the only source of human eTG (105). An alternative hypothesis is that eTG/IgA aggregates exists as circulating immune complexes. Accordingly, DH patients may show asymptomatic IgA immune complex deposition in the kidney (106); Preisz et al. described deposits of eTG/IgA1 complexes in both upper and deep dermal vessels in roughly 64% of DH patients (107); rare clinical manifestations of DH include digital purpura and ecchymosis, that show evidence of small vessels vasculitis on microscopic examination (49, 108); circulating eTG/IgA immune complexes can be found in patients with DH and their concentrations decrease under the GFD (109).

### Pathogenic Relevance of IgA Autoantibodies Against Epidermal Transglutaminase

A major controversial issue concerns the role of eTG/IgA aggregates in DH, since some studies clearly support their pathogenic relevance, whilst others did not (Table 2). For example, serum anti-eTG IgA autoantibodies has proven to correlate positively with disease activity (110): along with anti-tTG IgA and anti-endomysium autoantibodies (EmA), they significantly decrease in patients achieving clinical remission owing to the GFD, while increasing in patients who suffer a relapse (111). However, passive transfer of goat anti-eTG autoantibodies or DH human serum into SCID mice with human skin grafts fails to induce DH lesions, despite the formation of the typical granular eTG/IgA deposits into the engrafted skin (105, 112).

**Table 2:** Evidence which seem to support or point against the pathogenic relevance of eTG/IgA deposits which are typically found in the perilesional skin of patients with DH.

**Evidence supporting the pathogenic role of eTG/IgA aggregates in DH**
1) Circulating eTG IgA correlate with disease activity and disappear after a GFD
2) eTG/IgA complexes are enzimatically active, and activate fibrinogen at the tips of the papillary dermis
3) Circulating and skin resident neutrophils express Fc IgA receptor (CD89), suggesting a direct interaction between neutrophils and IgA.
**Evidence against the pathogenic role of eTG/IgA aggregates in DH**
1) eTG/IgA complexes can be found in the healthy skin of patients with DH
2) eTG/IgA complexes can be detected in the skin of coealic patients without DH
3) eTG/IgA complexes disappears even years after the introduction of the GFD and the resolution of the skin rash
4) Passive transfer of goat anti-eTG IgG or human DH sera in mice with human skin grafts reproduces DH-like granular deposits in the engrafted skin, but not DH lesions

*DH, dermatitis herpetiformis; eTG, epidermal transglutaminase; IgA, immunoglobulin A; GFD, gluten free diet*.

In the skin, eTG/IgA aggregates have been shown to activate fibrinogen, which can be found at the tips of the papillary dermis in a pattern similar to that of eTG/IgA aggregates (113). Fibrinolysis directly contributes to the blister formation in DH, and it is also believed to function as a chemoattractant for neutrophis, T-cells and macrophages, which are major components of the inflammatory infiltrate of DH (114). Both circulating and skin resident neutrophils in DH have been shown to highly express Fc IgA receptors, suggesting activation dependent on the interaction with the eTG/IgA complexes (115, 116). However, Donaldson et al. observed that granular IgA deposits could be also detected in the non-affected skin of DH patients (114). More intriguingly, Cannistraci et al. documented eTG/IgA co-localization in the papillary dermis, at the dermal-epidermal junction and in the vessel walls of coeliac patients without skin manifestations both before and during a GFD (10, 117, 118). It is also worth of mention that disappearance of eTG/IgA deposits from the skin occurs only in a subset of patients despite a long and strict GFD and no active skin rash, and takes much longer than serum autoantibodies or immune complexes (76, 119).

Recently, Taylor and Zone found that Potassium Iodide, a known precipitating factor of DH, increases the capacity of eTG/IgA complexes to bind the substrate cadaverin in normal skin cryosections from DH patients on dapsone or on a GFD. Thus, one could speculate that, during disease remission, eTG/IgA complexes preserve a baseline enzymatic activity, allowing tight binding with anchoring fibrils of the BMZ but not the activation of fibrinogen (76, 120).

### Cytokine Network in Dermatitis Herpetiformis and Mechanisms of Blister Formation

The mechanisms leading to tissue damage in DH are only partly understood. The microscopic finding of neutrophil accumulation at the papillary dermis and the responsiveness of skin lesions to dapsone support the key role of neutrophils in DH inflammation. Circulating neutrophils in DH show an increased expression of CD11b, decreased cell surface L-selectin, and increased Fc IgA receptor function, suggesting that they have already been primed before migrating into the skin (115). Indeed, neutrophils priming is likely to occur in the gut under the influx of gut-derived IL-8 (121). Accordingly, IL-8 mRNA was shown to be significantly increased in the small bowel of DH patients on a normal diet compared to that on a GFD. Circulating IL-8 decreases following the GFD, while persists elevated in DH patients who take a normal diet. A positive correlation between serum IL-8 and anti-tTG IgA antibodies has been also demonstrated, suggesting that the cytokine levels parallel the ongoing mucosal inflammation in the gut and depend directly on gluten ingestion (121). Likewise, the underlying mucosal inflammation leads to enhanced expression of the adhesion molecule E-selectin in endothelial cells from both lesional and non-lesional skin (122). Local production of cytokines and chemokines, including IL-8 and GM-CSF, in the presence of eTG/IgA deposits eventually allow migration of adhered neutrophils to the papillary dermis (121, 123). A recent study demonstrated a down-regulation of elafin, a serine protease inhibitor that inhibits a neutrophil mediated inflammatory response, in keratinocytes from DH skin. A similar finding could be also found in the epithelial cells from the small bowel of patients with active CD (124).

Activated neutrophils release neutrophil elastase and granzyme B, which induce subepidermal split by cleaving adhesion molecules of the BMZ, such as collagen VII (125). Accordingly, immunomapping studies have shown that dermal-epidermal detachment in DH mainly occurs within the lamina lucida, between collagen VII and laminin 332, and probably involves destruction of laminin 332 (126, 127). In addition, basal keratinocytes over-expresses collagenase, stromelysin-1, and urokinase-type plasminogen activator, which contribute to degrading basement membrane zone proteins (128).

The activation of the coagulation cascade is thought to be an additional pathomechanism of DH. Accordingly, Bognar et al. found a high prevalence of criofibrinogenaemia in untreated DH patients (129). Another study demonstrated an impaired fibrinogen/fibrin turnover in the disease. More in detail, untreated DH patients showed significantly prolonged clot lysis time and thicker fibrin fibers, which could be normalized by the *in vitro* adjunct of dapsone (130). Conversely, unlike BP, no studies demonstrated significant skin expression of tissue factor as well as elevation of serum D-dimer in patients with DH compared to healthy individuals (131, 132).

Previous studies have also suggested that other inflammatory cells participate to the pathogenesis of DH (133). In particular, T-lymphocytes, mononuclear phagocytes and B-cells were shown to accumulate around dermal vessels during the early phases of DH lesion formation (134). Other studies found increased Th2-related cytokines in the skin but not in the serum of DH patients (133, 135). By comparison, Makino et al. recently reported a significant elevation of IL-4, IL-5, IL-13, and eotaxin in fibrillary-type DH, whereas Th1-related cytokines, including IL-12 and IFN-γ, did not show significant differences compared to healthy controls (136).

In another study, our group demonstrated that, in DH skin, Tregs and IL-10 were significantly reduced compared to the skin of healthy subjects, whereas both coeliac and DH patients had a similar number of Tregs in duodenal biopsies, suggesting that the down-regulation of Tregs in the skin may be critical for the development of DH lesions (93).

### The Pathogenesis of Pruritus in Dermatitis Herpetiformis

While over the recent years different studies have gradually shed light on the major pathogenic mechanisms of DH, the pathogenesis of pruritus is far less clear. Probably, different pathways are involved, including neurogenic inflammation, mechanical itch dysesthesias, and release of inflammatory cytokines. Accordingly, Cynkier et al. demonstrated an over-expression of neuropeptides, including corticotropin releasing factor and the receptor for endotelin B in DH lesional skin, which may be released by activated keratinocytes (137). In DH, pruritus may be evoked or worsened by mechanical stimuli (allokinesis), such as clothing. Moreover, the intensity of the perceived pruritus is also enhanced (hyperkinesis).

Among inflammatory cytokines involved in pruritogenesis, IL-31 has gained a major interest. IL-31 belongs to the cytokine family of IL-6. IL-31 interacts with a heterodimeric receptor, which comprises the IL-31 receptor A (IL-31RA) and the oncostatin M receptor (138), and is expressed on various immune cells including T cells, keratinocytes, dendritic cells, eosinophils, basophils, macrophages, and dorsal root ganglia (139–145). In mice, over-expression of IL-31 was shown to evoke pruritus and induce inflammatory cells accumulation with increased number of mast cells. Injection of IL-31 in the dog also triggers a scratching behavior. Several studies have demonstrated an over-expression of IL-31 in the skin of different pruritic dermatoses, including atopic dermatitis (146), psoriasis (147), cutaneous T-cell lymphomas (148), nephrogenic pruritus (149), and mastocytosis (145).

IL-31 seems to be particularly implicated in pruritic dermatoses related to a prevalent Th2 type inflammation (150). A recent study demonstrated that eosinophils are the major source of IL-31 in BP, a prototype of highly pruritic Th2-mediated autoimmune blistering dermatoses (151). We have previously shown that Th2 type cytokines are also elevated in the skin of DH patients, thereby allowing speculation about a possible role of IL-31 in the associated pruritus. Interestingly, while a previous paper did not show significant elevation of serum IL-31 concentration in DH patients (152), a recent study by our group demonstrated that IL-31 was not only elevated in DH serum, but also significantly over-expressed in the skin, where it co-localized with IL-31RA (153).

Intriguingly, a monoclonal antibody targeting IL-31 is currently under clinical investigation in atopic dermatitis (154). If the role of IL-31 in DH pruritus were to be confirmed, IL-31 monoclonal antibodies will open interesting therapeutic perspectives, potentially allowing a faster control of DH pruritus, which is typically refractory to either topical or systemic treatments and improve only after months following a GFD.

## Gluten and the Skin: Not Always Dermatitis Herpetiformis

Although DH is the specific cutaneous manifestation of CD, several other skin diseases secondary to gluten ingestion are increasingly reported, especially in the last years, when the focus on gluten intolerance has grown steadily. In fact, despite the exclusion of gluten from the diet is even becoming a fashion, data from the literature demonstrate a higher incidence of skin diseases in patients with gluten-related disorders (14).

In 2012, Sapone et al. reviewed the spectrum of gluten-related disorders, including CD, DH, wheat allergy, gluten ataxia, and non-celiac gluten sensitivity (NCGS) (155). In their review, the authors highlighted that gluten ingestion could cause the involvement of different organs (such as bowel, nervous system, and skin), via the activation of different pathogenic mechanisms.

Accordingly, in recent years, several studies focused on skin manifestations different from DH that occurred in patients with both CD and NCGS.

### The Skin in Celiac Patients

According to the classification proposed by Humbert et al. (156), our group recently reviewed in detail the cutaneous and mucosal manifestations associated with CD (43). As a result, different groups of CD-related skin diseases were identified; however, the main distinction was between those that improve after a GFD and those that are just occasionally associated with CD.

In general, common dermatological diseases such as psoriasis, atopic dermatitis, urticaria, aphtous stomatitis, and rosacea are more frequently diagnosed in celiac patients than in the general population (157). Notably, their diagnosis is often difficult because of atypical clinical presentation, and their course is sometimes characterized by the resistance to standard therapies and the response (or at least the improvement) after the introduction of a GFD.

In this regard, the relationship between psoriasis and CD has been studied in depth, and patients with psoriasis seem to have a 3-fold increased risk of CD (158). Moreover, in a recent meta-analysis, patients with psoriasis were demonstrated to have an increased risk of positivity for serologic markers of CD and GFD was suggested to be of potential benefit for celiac antibody-positive patients with psoriasis (159).

Despite the association between the aforementioned skin diseases, testing patients having psoriasis, atopic dermatitis, or other dermatologic diseases for CD is not advisable, since the relative risk is low. There are only few conditions in which the screening is recommended, such as type 1 diabetes mellitus, autoimmune thyroiditis, autoimmune liver disease, juvenile chronic arthritis, Down syndrome, Turner syndrome, Williams syndrome, IgA deficiency, as well as patients having first-degree relatives with CD (160); among them, no skin disorders have been included yet.

### Non-celiac Gluten Sensitivity: The Skin as a Major Target

NCGS is a new entity within the group of gluten-related disorders that is being increasingly reported in the medical literature. Despite the lack of specific biomarkers, that makes the diagnosis of NCGS one of exclusion, the reported high frequency of the disease and its potential impact on the quality of life make NCGS a health challenge.

NCGS mainly affects the bowel; however, extra-intestinal manifestations are common, being the skin one of the major target of the disease.

To date, only two studies have investigated the cutaneous manifestations of NCGS, including one by our group. In general, skin lesions were found to be predominantly located on the extensor surfaces of the upper and lower limbs, and consisted of erythema, papules, crusts, and sometimes, vesicles, resembling subacute eczema, or even DH. Some patients had hyperkeratotic scales overlying mild erythematous plaques similar to psoriasis, while others showed urticarial plaques or wheals (161, 162). Besides the prevalent clinical phenotype, all the patients suffered from intense pruritus, that in about 10% of the cases represented the only symptom.

Interestingly, in both studies, patients were shown to respond well to a GFD, with some patients achieving disease remission within only 1 month following the GFD (161). It is worth mentioning that about 80% of the patient included in our study showed at DIF the presence of DH-like granular C3 deposits at the dermal-epidermal junction, but none showed the presence of IgA.

This immunopathologic profile, together with itching and the resolution of the skin lesions after the introduction of a GFD were considered as the main features of this new entity, referred to as “cutaneous gluten sensitivity.”

Despite the low number of enrolled patients, suggesting that further studies are required to confirm these data, the introduction of the concept of specific skin manifestations related to NCGS may be helpful for the management of the patients, as is for DH and CD.

In fact, the main issue of NCGS is represented by the diagnosis, that relies on a procedure that is impractical to be implemented in the clinical setting (i.e., double blind placebo controlled challenge with duodenal biopsy) (163). Accordingly, the identification of specific skin features such as cutaneous gluten sensitivity might allow the diagnosis of NCGS without the need of invasive investigations.

## Diagnosis of Dermatitis Herpetiformis

DH is a difficult disease to be diagnosed. Accordingly, the delay from the occurrence of the first symptoms or clinical signs to the diagnosis is usually of several months or even years, although it is reported to be decreasing in the last decades, probably due to a major awareness both of DH itself and of coeliac disease (25).

This diagnostic delay is caused by the rarity of DH and the polymorphic cutaneous manifestations, which can be misdiagnosed as other chronic pruritic dermatoses including autoimmune blistering diseases such as BP or Linear IgA bullous dermatosis but also atopic dermatitis, eczema, prurigo, urticaria, or scabies (7). Moreover, in the last years skin manifestations of NCGS have emerged as a novel diagnostic challenge in patients with gluten intolerance, since they can clinically resemble DH and can share similar intestinal involvement (161, 162).

### The Role of Skin Biopsy: Are IgA Deposits Still Pathognomonic of Dermatitis Herpetiformis?

After taking patient's history and clinical examination, the first step to make a diagnosis of DH is to perform a skin biopsy. The biopsy specimen can be investigated for histopathological examination, which may show typical features such as subepidermal vesicles and blisters associated with accumulation of neutrophils at the papillary tips (7). However, in a third of the cases (164), histopathology is non-specific; atypical findings have been also reported, including acantholysis, leading to a possible histopathologic overlap with pemphigus (165).

**Figure 4:**
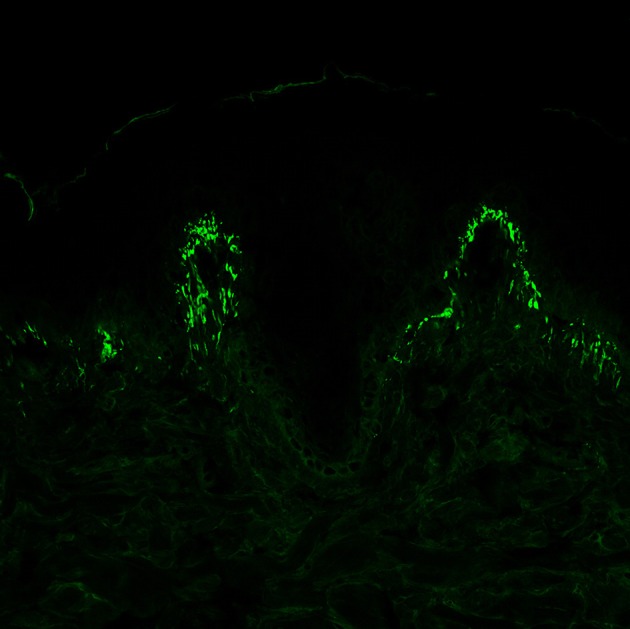
Direct immunofluorescence of perilesional skin specimens from patients with dermatitis herpetiformis (DH). Granular IgA deposits at the dermal papillary tips are considered a pathognomonic finding of DH (magnification 400×).

Therefore, although histopathological examination may be helpful, DIF from the perilesional skin is still considered the gold standard for the diagnosis. The main finding is the presence of granular IgA deposits at the dermal papillae and/or at the dermal-epidermal junction (Figure 4) (7); in some cases, and in up to 50% of Japanese patients (79), a fibrillar deposition of IgA could be found.

Besides IgA, other immunoreactants can be found at the dermal-epidermal junction or at the dermal papillae in the perilesional skin. Among them, IgG granular deposits are infrequently detected, while IgM and C3 granular deposits are present in more cases.

In some cases, patients may show only granular deposition of C3 in the absence of IgA, IgG, or IgM. Recently, a case series of 20 patients showing these findings at DIF has been reported (166). Half of them had clinical features mimicking DH and very few of them showed low titres of anti-eTG, anti-tTG, anti-gliadin, and anti-BP180 antibodies. The authors proposed the term granular C3 dermatosis to describe this condition (166), that might be considered as an umbrella concept including different diseases (DH, cutaneous gluten sensitivity, non-DH dermatoses occurring in patients with CD) rather than a distinct clinical entity.

Although its prominent role in the diagnostic algorithm for DH, due to its high sensitivity and specificity, DIF can have some limitations. As an example, several papers reported patients diagnosed with DH according to compatible clinical features, histopathology, concomitant presence of CD, the response to the GFD or dapsone, and the recurrence of cutaneous lesions after a gluten challenge, but showing negative DIF results (167, 168).

In some instances, DIF may result negative because of the site of the biopsy; in fact, when the skin specimen is taken in lesional skin or too far from the skin lesions, it may lack the presence of IgA deposits (7). However, in some confirmed DH cases, even repeated biopsies performed at different times gave negative DIF results, suggesting that false-negative patients are possible (169, 170).

Another pitfall can be related to the interpretation of DIF. On this point, a recent paper from our group reported a case-series of six celiac patients presenting with skin diseases different from DH, such as contact eczema, dermatophytosis, granuloma annulare, pytiriasis rosea, lichenoid dermatitis, and psoriasis. Notably, all these patients showed a granular deposit of IgA at the dermal-epidermal junction and/or at the papillary tips (10). As previously mentioned, Cannistraci et al. observed granular IgA deposits in the healthy skin of nine celiac patients without any cutaneous manifestation (117). Both these studies raise the hypothesis that granular IgA deposits may occur also as a cutaneous marker of CD. Interestingly, while in study by Cannistraci et al. coeliac patients showed IgA/eTG co-localization in the granular deposits, no IgA/eTG co-localization was documented in any case from our study.

To conclude, the diagnosis of DH should be the result of an overall assessment, including clinical, histological, and immunopathological findings and could not rely only on DIF findings. Whether assessing the co-localization between IgA and eTG deposits in the perilesional skin might be helpful to confirm the diagnosis of DH in doubtful cases warrants further investigation.

### Beyond Direct Immunofluorescence: Serology and Other Investigations

Among other examinations that can help in the diagnosis of DH, serology plays a primary role, mainly as a screening tool. In fact, as happens for CD, patients with DH test positive in the majority of cases for anti-tTG, anti-EMA and anti-deamidated synthetic gliadin-derived peptides, that overall showed a sensitivity ranging from 50 to 95%, and a specificity higher than 90% (171). Moreover, in the last years, after the demonstration of eTG as the main autoantigen for DH, anti-eTG antibodies have been shown to be a promising tool for the diagnosis of DH (12, 104, 111). Unfortunately, although their sensitivity and specificity for DH are close to those of anti-tTG antibodies, anti-eTG antibodies can be found in about 30 to 50% of the patients with CD (12, 172), and therefore, are not helpful to differentiate between DH and other dermatoses occurring in celiac patients.

Other antibodies have been tested in patients with DH showing promising results, such as anti-neo epitope tissue TG (173) and anti-GAF3X antibodies (174); however they were no further tested and their high sensitivity and specificity values should be confirmed.

A comprehensive assessment of patients with DH can include other investigations that may be helpful in doubtful cases. Duodenal biopsy is usually not required but, since it is the gold standard for the diagnosis of CD, it should be performed if the clinical picture or other findings are highly evocative for the diagnosis of DH but DIF results negative (7). In that case, the presence of villous atrophy at histopathology is important to confirm (or to exclude) the diagnosis of CD and to support the introduction of a GFD, at least in the Caucasians. Accordingly, it is worth noting that duodenal biopsy might be not helpful in Japanese DH patients, because of the weaker association of DH with CD in that population and because diseases other than CD represent a more frequent cause of villous atrophy in Asia, including tropical sprue, parasitic infections, immunoproliferative small intestinal diseases and combined variable immunodeficiency disease (175).

Since virtually all the patients with CD and DH, at least in western countries, have HLA-DQ2 or DQ8 aplotypes, HLA testing may be helpful for its high negative predictive value in order to exclude a diagnosis of DH and to avoid repetition of unnecessary investigations (171).

Finally, in the last years, other potential diagnostic tools have been proposed for DH. As an example, dermoscopy was found to be helpful in the differential diagnosis of autoimmune bullous diseases involving the scalp. In particular, patients with DH usually show the absence of yellow scales, that are more typical of pemphigus patients, but display extravasation and clustered dotted vessels (176), that seem to be a specific finding of the disease and are found even in the petechial lesions of palms and soles (177).

Other more complex investigations, such as the analysis of microbiota and of metabolic signature of the patients, may provide some information on the patients with DH. However, although they were investigated in detail in patients with CD, paving the way for their potential use in the clinical setting (178, 179), very scarce data are available for DH (180), and further studies are needed to address the questions that are still open in this field.

## Treatment

### The Central Role of Gluten: What's Beyond Gluten-Free Diet?

Since DH is the specific cutaneous manifestation of CD, at least in the Caucasians, all the patients require a GFD. Although some authors suggested that a subgroup of the patients with DH can go into remission and, therefore, gluten-free diet may be stopped (13), other studies demonstrated that DH is associated with an increased risk of associated diseases, such as non-Hodgkin lymphomas (28). Moreover, although this risk is higher in the first 5 years after the diagnosis, it overlaps that of the general population thereafter (32). As a result, since GFD in DH is a way to prevent complications rather just a symptomatic treatment for skin lesions, it should be maintained for all the life (181).

Gluten-free diet requires strict monitoring of all ingested foods, it is time-consuming, and socially restricting (182), and patient compliance depends on different individual and environmental factors, with a self-reported diet adherence in adult patients with CD ranging from 36 to 96%(183). By contrast, patients with DH seem to have a better compliance to gluten-free diet, with an overall adherence of 98% (percentage that decreases to 72% for strict adherence) (32). However, since gluten contamination is possible even in supposed gluten-free food, nutritional monitoring, and participation in support groups are recommended for both patients with CD and DH (184). Moreover, some patients that adhere to gluten-free diet are refractory even several years after the introduction of the diet (185).

According to the limitations of gluten-free diet reported above, novel approaches to treat patients with CD and DH are currently under investigation. Such new therapies rely on blocking at different steps the pathogenic process occurring in gluten-sensitive disorders.

For example, the reduction of gluten immunogenicity via the production of genetically modified grains may lead to a decrease of the number of patients that are able to develop autoimmunity to gliadin-tissue TG complexes (186). RNA interference is another way that could be applied in order to reduce gluten toxicity (187). Moreover, gluten exposure could be reduced using some binders that allow its sequestration in the bowel lumen, or by hydrolysis of gluten peptides that are resistant to proteolysis in a physiological setting using orally administered glutenases (188). Finally, the exposure to immunogenic peptides can be prevented by altering gut permeability; in this regard, the blockade of zonulin, a tight junction protein that regulates the epithelial transit of molecules, with inhibitors such as larazotide has proven to ameliorate symptoms of patients with CD (189).

Other steps that can be targeted in patients with CD are related to the activation of the immune response. tTG activity inhibition, HLA-DQ2 blocking with gluten peptide analogs or immune tolerance induction using specific gluten epitopes are under study or are being tested in patients with CD (190, 191).

### Pharmacologic Treatment of DH: When Gluten-Free Diet Is Not Enough

All the approaches reported above are being developed for patients with CD but, since they are oriented to prevent gluten sensitization or to restore gluten tolerance, they might work even for DH. The latter, however, has some specific therapeutic issues that differ from those of CD. In fact, while intestinal symptoms usually resolve in few weeks, cutaneous manifestations may last months or even years after the introduction of a GFD. Therefore, in most cases, pharmacologic treatment is required in order to control itching as well as the skin rash (7).

Despite the lack of randomized controlled trials, dapsone has been considered the first line treatment in patients with DH for over 70 years. Dosages of about 50 to 100 mg per day are usually sufficient to clear the skin rash. At these dosages, side effects are usually rare. However, hemolytic anemia and methemoglobinemia, that are dose-dependent side effects, can be seen in some patients. As a result, all the patients taking the drug should be followed up with frequent testing of hemoglobin levels and reticulocytes. In patients with glucose-6-posphate dehydrogenase deficiency dapsone treatment is contraindicated (7).

In two case reports, topical dapsone 5% gel was also shown to be effective as an adjuvant treatment for DH, which is not adequately controlled by GFD or oral dapsone (192, 193). Compared to the oral counterpart, topical dapsone shows a lower incidence of side effects and can be administered safely in patients with glucose-6-posphate dehydrogenase deficiency (192).

Possible alternatives to dapsone are the so-called sulfonamides, including sulfasalazine, sulfapyridine, and sulfamethoxypyridazine, that may potentially cause hemolytic anemia and gastrointestinal upset, but require a less strict monitoring than dapsone (7). Furthermore, a recent report suggested that combination therapy with dapsone and sulfasalazine may be effective in patients who do not tolerate increasing doses of dapsone monotherapy (194).

Several other pharmacologic treatments have been proposed for DH. Case reports do exist showing that cyclosporin A, azathioprine, colchicine, heparin, tetracyclines, nicotinamide, and mycophenolate may control the acute rash in patients with DH (171). Among them, some authors suggested that colchicine may be used as a second choice after dapsone, due to its antineutrophilic and antithrombotic activity (195), since DH was reported to be associated with a decreased fibrinolytic potential (130).

Besides these “old” drugs, biologics may become the next step in DH pharmacologic treatment. Among them, rituximab has been proven to be effective in a patient resistant to GFD, dapsone, sulfasalazine, and conventional immunosuppressive agents such as azathioprine, and was suggested as a viable treatment option for recalcitrant DH (196).

### Celiac Disease and Dermatitis Herpetiformis: Is Prevention Possible?

In the last years, some authors focused on the risk factors associated with the development of CD, aiming at the prevention of the disease in genetically at-risk infants or children. Randomized controlled trials investigated the possible role of the age at which gluten is introduced, showing that neither ingestion of small gluten amounts between weeks 16–24, nor delayed introduction of gluten (at 6 or at 12 months) modified the incidence of CD in the studied groups (197, 198).

Besides gluten, microbiota has been suggested to act as a trigger in several autoimmune diseases, including CD. In the latter, both genetic and environmental factors, such as rotavirus infection, may modified the gut microbiota of at-risk patients that, in turn, can affect bowel immunity and permeability (199). In particular, altered gut microbiota increases the production of pro-inflammatory cytokines, impairs the mucosal barrier, and produces microbial TG (199), that is a target of antibodies found in celiac patients (200). Therefore, targeting the gut microbiota via probiotics may be a reasonable approach in order to prevent CD in at-risk individuals, and trials are currently underway (199).

Notably, all these data are available for CD. However, due to the close correlation with CD, it could be assumed that such strategies may work even for the prevention of DH, and studies on these topics would be advisable.

## Final Considerations and Future Perspectives

DH is an autoimmune bullous disease associated with a chronic, usually asymptomatic, autoimmune enteropathy, that arises in genetically susceptible individuals, in the presence of gluten proteins consumed in common grain products.

One of the main concerns of DH is still represented by misdiagnosis, due to its rarity, the growing report of atypical clinical presentations and the possible occurrence of IgA deposits in non-DH skin diseases. In the near future, the diagnostic challenge is expected even to increase, due to the falling incidence of DH (16). This is reflected by the quite long diagnostic delay, found even in high prevalence areas (25).

In the last years, NCGS has emerged as a new entity within the spectrum of gluten-related disorders. Similarities in both clinical and immunopathological findings may enhance the diagnostic challenge and have important therapeutic implications. In fact, although GFD is the first line treatment for both DH and NCGS, patients with DH should be followed up closely for dietary adherence, nutritional deficiencies, and potential complications.

Recent advances in the pathogenesis of DH have paved the way for the development of new treatments to be used in the time window between the beginning of the GFD and the complete resolution of skin lesions. As an example, the recent finding of elevated serum levels of IL-17 and IL-36 in DH patients supported their possible role in the activation of neutrophils and NK cells, making them as possible targets for new therapeutic strategies (201). In addition, the involvement of IL-31 pathway in DH suggests a connection among the immune system, the nervous system and itch, and its targeting holds promise for the treatment of the patients (153).

## Author Contributions

EA, RM, and MC designed the study and drafted the manuscript. LQ, AV, DB, and VB drafted the manuscript. EA, RM, and MC revised the whole manuscript. All the authors approved the final content of the manuscript.

### Conflict of Interest Statement

The authors declare that the research was conducted in the absence of any commercial or financial relationships that could be construed as a potential conflict of interest.
